# Levosimendan and mortality after coronary revascularisation: a meta-analysis of randomised controlled trials

**DOI:** 10.1186/cc10263

**Published:** 2011-06-08

**Authors:** Ritesh Maharaj, Victoria Metaxa

**Affiliations:** 1 Kings College Hospital, Denmark Hill, London, SE5 9RS, UK

## Abstract

**Introduction:**

Patients undergoing coronary revascularization often require inotropic support that has been associated with an increased risk for death and morbidity. The purpose of this study was to evaluate the effect of levosimendan versus control on survival after coronary revascularization.

**Methods:**

A systemic review and meta-analysis of the literature was carried out on published randomized controlled clinical trials that investigated the efficacy of levosimendan compared to other therapy in patients having coronary revascularisaion. The databases searched were Pubmed, EMBASE, the Cochrane Registry of Clinical Trials and the metaRegister of Controlled Trials. Studies that compared levosimendan to any other therapy for coronary revascularisation in adult humans and reported at least one outcome of interest were considered for inclusion. Both percutaneous coronary intervention and cardiac surgery were included. Data extraction was performed independently by two reviewers using predefined criteria. Relevant outcomes included mortality, cardiac index, cardiac enzymes, length of stay and post-procedural atrial fibrillation.

**Results:**

The meta-analysis included 729 patients from 17 studies. Levosimendan was associated with a mortality reduction after coronary revascularization, (19/386 in the levosimendan group vs 39/343 in the control arm) odds ratio (OR) 0.40 (95% confidence interval (CI) 0.21 to 0.76, *P *for overall effect 0.005, *P *for heterogeneity = 0.33, I^2 ^= 12% with a total of 729 patients. Levosimendan also had a favourable effect on cardiac index (standardised mean difference 1.63, 95% CI 1.43 to 1.83, *P *for overall effect < 0.00001), length of intensive care stay (random effects model, mean difference - 26.18 hours 95% CI 46.20 to 6.16, *P *for heterogeneity < 0.00001, I^2 ^= 95%, *P *for overall effect *P *= 0.01), reductions in the rate of atrial fibrillation (OR 0.54, 95% CI 0.36 to 0.82, *P *for effect = 0.004, *P *for heterogeneity 0.84, I^2 ^= 0% for 465 patients) and troponin I levels group (mean difference -1.59, 95% CI 1.78 to 1.40, *P *for overall effect < 0.00001, *P *for heterogeneity < 0.00001, I^2 ^= 95%). Limitations of this analysis are discussed.

**Conclusions:**

Levosimendan is associated with a significant improvement in mortality after coronary revascularization. There are also improvements in several secondary endpoints. A suitably powered randomised controlled trial is required to confirm these findings and to address the unresolved questions about the timing and dosing of levosimendan.

## Introduction

Following coronary revascularisation, patients are still vulnerable to a low cardiac output state and tissue hypoperfusion. Some patients may require bridging therapy in order to realise the benefits of revascularisation. There is substantial geographic variation, but it is estimated that between 8 and 25% of patients undergoing coronary revascularisation require inotropic support for myocardial dysfunction [1-3]. This group of patients carry a substantial burden of morbidity and mortality [4]. Pharmacologic support is commonly limited to catecholaminesand phosphodiesterase III inhibitors (PDEIs) with levosimendan gaining prominence [5]. There is a lack of suitably powered randomised control trails to guide the choice of inotrope in this group of patients. Both catecholamines and PDEIs are associated with increased post-operative myocardial oxygen consumption and arrythmogenesis [6]. Additionally catecholamines have also been associated with impaired coronary vasodilatatory reserve [7].

Levosimendan sensitises myofilaments to calcium by binding to the calcium saturated troponin C, increasing their load-independent contraction [8]. The pleiotropic effects of levosimendan include vasodilatation by opening ATP-sensitive potassium channels (K_ATP_) in vascular smooth muscle as well as in mitochondrial K_ATP _channels.

Early clinical trials, though underpowered, have suggested that levosimendan would be useful in the setting of post coronary revascularisation myocardial dysfunction. More recently there have been several completed clinical trials in this group of patients. The principal objective of this study was to evaluate the association between levosimendan, compared with conventional therapy on mortality, in randomised control trialsin patients having coronary revascularisation.

## Materials and methods

### Search strategy

The primary search for randomised clinical trials (RCTs) was conducted using Pubmed, EMBASE and the Cochrane Registry of Clinical Trials. The search term used was 'levosimendan'. The search was combined with filters to identify RCTs in the Pubmed and EMBASE database and is available as supplementary material (see Additional file 1 for details). We also searched the metaRegister of Controlled Trials using the term 'levosimendan' [9]. No language restriction was placed but the search was limited to adult human subjects. We used backward snowballing by reviewing the bibliographies of included RCTs and review articles to identify otherwise unrecognised publications. The electronic database search included publications from 1966 and was finalised on 31 August 2010. The study did not require ethical approval.

### Study selection

The authors reviewed all abstracts to identify potential RCTs in a standardised manner. The inclusion criteria were reports of RCTs that compared levosimendan to any other therapy for coronary revascularisation in adult humans and reported at least one outcome of interest. Relevant outcomes included mortality, haemodynamic parameters (for example, cardiac index), cardiac enzymes, length of stay and post-procedural atrial fibrillation. If the abstract suggested that the study could potentially meet the inclusion criteria, the full text article was then retrieved and reviewed. Disagreements between reviewers were resolved by consensus.

### Data abstraction and study characteristics

All included studies were assessed for internal validity and risk of bias according to the Cochrane Collaboration methods. Each report was assessed for the adequacy of allocation concealment, blinding, performance of an intention to treat analysis and the extent of loss to follow-up.

Data included baseline patient characteristics including age and co-morbidity, baseline left ventricular ejection fraction, details of levosimendan dose and specifics of comparator therapy, type of coronary revascularisation, haemodynamic and clinical outcome data. When data was missing attempts were made to contact the authors.

### Data synthesis and analysis

Agreement on inclusion of studies was assessed using the kappa statistic. Heterogeneity was measured using the Cochrane Q test and quantified with the *I^2^*statistic. An *I^2^*value of > 50% was considered at least moderate heterogeneity.

Data from individual studies were collected to allow calculation of odds ratio (OR). The primary analysis was conducted by means of the Peto fixed effects method when I^2 ^< 25% and with the random effects model when I^2 ^> 25%. Weighted mean difference (WMD) and 95% CI were calculated for continuous variables (haemodynamics and troponin I). The potential for bias was assessed by visual inspection of funnel plots. Sensitivity analyses were conducted by comparing the fixed and random effects models as well as by evaluating the risk of mortality in studies with a low risk of bias only. The analysis was performed using statistical software SPSS 16 (SPSS Chicago, IL, USA) and Revman 5.0 (available from the Cochrane Collaboration Group). The study was performed in compliance with the Preferred Reporting Items for Systematic Reviews and Meta-analysis (PRISMA) statement [10,11].

## Results

**Figure 1:**
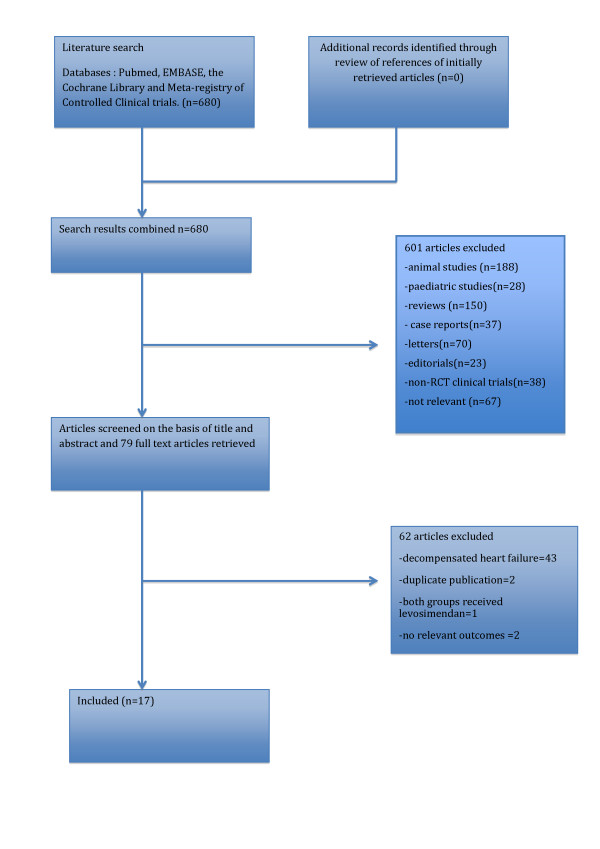
**Flow diagram of literature search and selection process of the studies**.

There were 680 reports identified by the search, 79 full text articles retrieved for in depth review. We identified 17 eligible studies involving 729 patients that were included in the final analysis. Figure 1 describes the flow and reasons for exclusion. Agreement on study inclusion was reached in 76 of the 79 cases (kappa = 0.92). The characteristics of the included studies are described in Table 1. An appraisal of study quality and risk of bias is presented in Table 2. Overall studies were of variable quality and many reports lacked relevant details to assess selection, performance or reporting bias. A risk of bias graph is included in the supplementary material (see Additional file 2 for details).

**Table 1:** Description of the studies included in the meta-analysis

Source	Year	Patients	Mean Age (years ± SD)		Setting	Time of administration	Mean Baseline LVEF % ± SD		Control	Follow-up
			**Levosimendan**	**Control**			**Levosimendan**	**Control**		

**Al-Shawaf *et al***. [35]	2006	30	60.5 ± 1 1	58 ± 10	Type 2 diabetes with LCOS after CABG.	Post intervention	29 ± 6	31 ± 6	Milrinone	Hospital stay

**Alvarez *et al***. [36]	2005	30	71.5 ± 5.2	71.9 ± 5.2	Elective CABG with LCOS	Post intervention	35 ± 5	37 ± 4	Dobutamine	Hospital stay

**Alvarez *et al***. [37]	2006	50	71.2 ± 7.2	67.0 ± 8.0	Elective CABG with LCOS	Post intervention	35 ± 4	34 ± 5	Dobutamine	Hospital stay

**Barisin *et al***. [38]	2004	33	62.4 ± 7.1	57.9 ± 10.4	Elective OPCAB with good LV function	Pre- intervention	62 ± 8	58 ± 10	Placebo	Hospital stay

**De Hert *et al***. [39]	2007	30	67 ± 11	69 ± 10	Elective CABG	Intraoperatively	24 ± 6	27 ± 3	Milrinone	Hospital stay

**De Hert *et al***. [40]	2008	60	67.5 ± 9.5	69 ± 10	Elective CABG	Inraoperatively	22 ± 5	25 ± 3	Milrinone	Hospital stay

**De Luca *et al***. [41]	2005	26	58.6 ± 8.7	57.1 ± 9.3	Acute MI undergoing PCI	Post intervention	28 ± 3	30 ± 3	Placebo	Hospital stay

**Eriksson *et al***. [42]	2009	60	64 ± 10	64 ± 10	Elective CABG with LVEF<50%	Intraoperatively	36 ± 8	36 ± 8	Placebo	Hospital stay

**Fuhrmann *et al***. [33]	2008	32	68 ± 74	68 ± 8.1	Cardiogenic shock after acute MI	Post intervention	22 ± 9	27 ± 10	Enoximone	Hospital stay

**Husedzinovic *et al***. [43]	2005	24	61 ± 5.4	61 ± 5.3	Elective OPCAB with normal LVEF	Pre- intervention	56 ± 5	59 ± 2	placebo	Hospital stay

**Jarvela *et al***. [44]	2008	24	69 ± 11	67 ± 10	Elective AVR and CABG	Intraoperatively	50 ± 4	65 ± 5	Placebo	Hospital stay

**Levin *et al***. [45]	2008	137	62.4	61.7	LCOS after elective CABG	Post intervention	37 ± 4	38 ± 5	Dobutamine	Hospital stay

**Lilleberg *et al***. [46]	1998	23	56.9 ± 7.9	59 ± 5.7	Elective low risk CABG	Post intervention	61 ± 9	58 ± 9	Placebo	Hospital stay

**Samimi-Fard *et al***. [47]	2008	22	65 ± 12	63 ± 11	Acute MI with Cardiogenic shock after PCI	Post intervention	29 ± 2	30 ± 3	Dobutamine	Two years

**Sonntag *et al***. [48]	2004	24	60	60	PCI after acute MI	Post intervention	58 ± 3	62 ± 4	Placebo	Hospital stay

**Tritapepe *et al***. [49]	2006	24	66.5 ± 5.9	69.5 ± 5.6	Elective CABG	Pre- intervention	50 ± 7	52 ± 5	Placebo	Hospital stay

**Tritapepe *et al***. [1]	2009	106	66.5 ± 7.8	64. ± 8	Elective CABG	Pre- intervention	44 ± 10	42 ± 11	Placebo	Hospital stay

CABG, coronary artery bypass graft; LCOS, low cardiac output state; LVEF, left ventricular ejection fraction; MI, myocardial infarction; OPCAB, off pump coronary artery bypass graft; PCI, primary coronary intervention.

**Table 2:** Risk of bias assessment of included studies

Source	Adequate sequence generation	Allocation concealment	Blinding	Incomplete outcome data addressed	Free of selective reporting	Free of other Bias	Concurrent therapies similar	Overall risk of bias
**Al-Shawaf *et al***. [35]	Unclear	Yes (sealed envelopes)	No	Unclear	Yes	Yes	Yes	Moderate
**Alvarez *et al***. [36]	unclear	Unclear	No	Unclear	Yes	Yes	Yes	Moderate
**Alvarez *et al***. [37]	unclear	Unclear	No	Unclear	Yes	Yes	Yes	Moderate
**Barisin *et al***. [38]	Yes (computer generated)	Yes	Yes (patients, doctors, adjudicators)	Unclear	Yes	Yes	Yes	Low
**De Hert *et al***. [39]	Yes (computer generated)	Yes (sealed envelopes)	Yes (adjudicators)	Unclear	Yes	Yes	Yes	Low
**De Hert *et al***. [40]	Yes (computer generated)	Yes (sealed envelopes)	Yes(adjudicators)	Unclear	Yes	Yes	Yes	Low
**De Luca *et al***. [41]	Unclear	Unclear	No	Unclear	Yes	Yes	Yes	Moderate
**Eriksson *et al***. [42]	Yes (permutated bocks)	Yes	Yes (patients, doctors)	Unclear	Yes	Yes	Yes	Low
**Fuhrmann *et al***. [33]	Yes (computer generated)	Yes (computer generated)	Yes (patients doctors)	Unclear	Yes	Yes	Yes	Low
**Husedzinovic *et al***. [43]	Yes (casting lots)	Yes	Yes (patients, adjudicators)	Unclear	Yes	Yes	Yes	Moderate
**Jarvela *et al***. [44]	Yes (computer generated)	Yes (sealed envelopes)	Yes (patients, doctors, adjudicators)	Unclear	Yes	Yes	Yes	Low
**Levin *et al***. [45]	Yes (computer generated)	Unclear	No	Unclear	Yes	Yes	Yes	Moderate
**Lilleberg *et al***. [46]	Unclear	Unclear	Yes (patients, doctors, adjudicators)	Unclear	Yes	Yes	Yes	Moderate
**Samimi-Fard *et al***. [47]	Unclear	Unclear	No	Unclear	Yes	Yes	Yes	Moderate
**Sonntag *et al***. [48]	Unclear	Yes	Yes (patients, doctors, adjudicators)	Unclear	Yes	Yes	Yes	Moderate
**Tritapepe *et al***. [49]	Yes (computer generated)	Unclear	Yes (Patients, physicians, adjudicators)	Unclear	Yes	Yes	Yes	Low
**Tritapepe *et al***. [1]	Yes (computer generate)	Unclear	Yes (patients, physicians, adjudicators)	Unclear	Yes	Yes	Yes	Low

**Figure 2:**
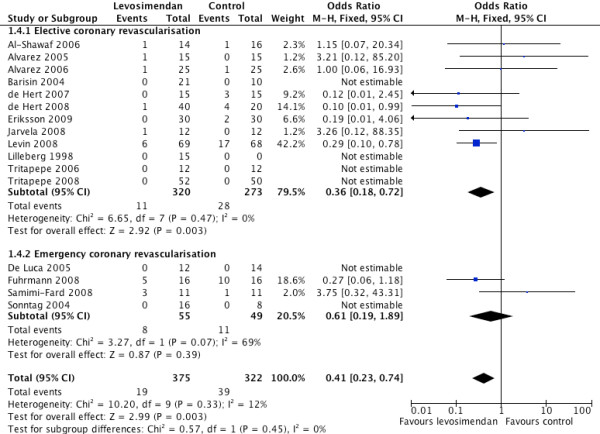
**Forest plot for risk of mortality with subgroups elective and emergency revascularisation**.

Analysis of the data showed that, compared with a control intervention, levosimendan was associated with a significant mortality reduction, (19/387 in the levosimendan group vs 39/342 in the control arm) OR 0. 41 (95% CI 0.23 to 0.74, *P *for overall effect 0.005, *P *for heterogeneity = 0.33, I^2 ^= 12% with a total of 729 patients) (Figure 2). A subgroup analysis comparing the effect of levosimendan on mortality in elective versus emergency coronary revascularisation was performed. The elective revascularisation showed a statistically significant mortality benefit compared with the emergency revascularisation group (OR 0.36, 95% CI 0.18 to 0.72, *P *for overall effect = 0.003, I^2 ^= 0% compared with OR 0.61, 95% CI 0.19 to 1.89, *P *for overall effect = 0.39, I^2 ^= 69% respectively). All the emergency revascularisations were PCI. The overall result was consistent when a random effects model was used (OR 0.43 (95% CI 0.21 to 0.89)).

**Figure 3:**
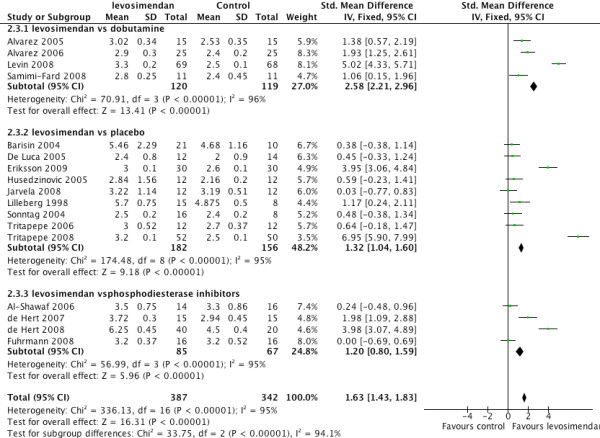
**Forest plot for cardiac index with sub-groups dobutamine, placebo and phosphodiesterase inhibitors**.

Levosimendan had a favourable effect on cardiac index (standardised mean difference 1.63, 95% CI 1.43 to 1.83, *P *for overall effect < 0.00001) although there was significant heterogeneity (*P *< 0.000001 I^2 ^= 95%). A sub-group analysis was undertaken to explore the heterogeneity. Subgroups were defined by the comparator drug, that is, the placebo, dobutamine or phosphdiesterase inhibitors. The sub-group analysis did not show any difference in treatment effect (Figure 3).

**Figure 4:**
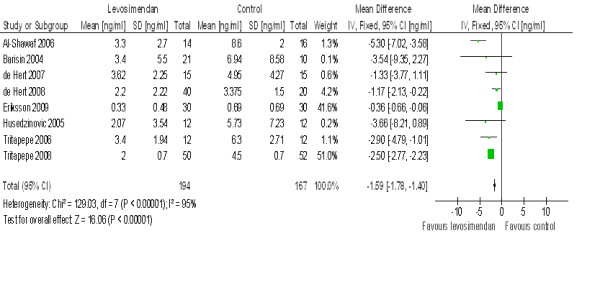
**Forest plot of comparison of levosimendan vs control with Troponin I level as the outcome measure outcome**.

Post revascularisation, troponin I levels were significantly higher in the control group compared with the levosimendan group (mean difference -1.59, 95% CI 1.78 to 1.40, *P *for overall effect < 0.00001, *P *for heterogeneity < 0.00001, I^2 ^= 95%) with 361 patients included (Figure 4).

**Figure 5:**
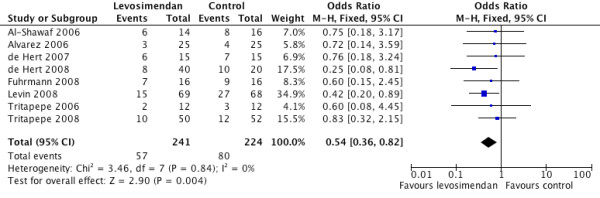
**Forest plot comparing rates of post revascularisation atrial fibrillation for levosimendan versus control**.

**Figure 6:**
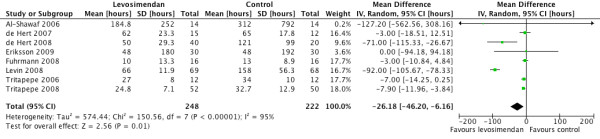
**Forest plot comparing length of stay in the levosimendan versus control groups**.

**Figure 7:**
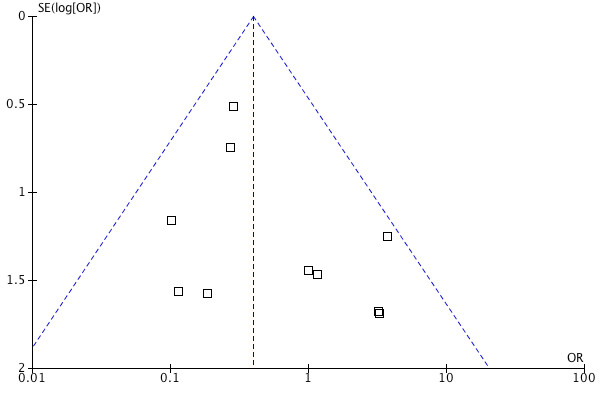
**Funnel Plot for risk of mortality including all studies**.

There was a significantly lower rate of post-revascularisation new-onset atrial fibrillation in the levosimendan treated group (OR 0.54, 95% CI 0.36 to 0.82, *P *for effect = 0.004, *P *for heterogeneity 0.84, I^2 ^= 0% for 465 patients (Figure 5). There was also a significant difference in the length of intensive care stay in favour of levosimendan compared with control (random effects model, mean difference - 26.18 hours 95% CI 46.20 to 6.16, *P *for heterogeneity < 0.00001, I^2 ^= 95%, *P *for overall effect *P *= 0.01) (Figure 6). The likelihood of small study bias robustness was evaluated by inspection of the funnel plot (Figure 7). Inspection of the funnel plot found no major evidence of such bias and added to the validity and robustness of the study. A sensitivity analysis that only included those studies with a low risk of bias (Table 1) found an OR of 0.25 (95% CI 0.09 to 0.68). A further sensitivity analysis to establish differential effects based on the comparator was performed. The largest effect size was observed in the levosimendan versus phosphodiesterase group (OR 0.23 (95% CI 0.08 to .65)). The levosimendan versus dobutamine group showed a strong trend in favour of levosimendan but was not statistically significant (OR 0.54 95% CI 0.25 to 1.17)). The levosimendan versus placebo group suggested a positive effect in favour of levosimendan but did not achieve statistical significance (OR 0.66 (95% CI 0.11 to 4.08)) (Table 3).

**Table 3:** A sensitivity analysis

	Number of studies	OR	95% CI	*P *for overall effect	I^2 ^%
Studies with low risk of bias	8	0.25	0.09 to 0.68	0.007	0
Levosimendan versus placebo	9	0.66	0.11 to 4.08	0.65	35
Levosimendan versus phosphodiesterase	4	0.23	0.08 to 0.65	0.003	0
Levosimendan versus dobutamine	4	0.54	0.25 to 1.17	0.12	43

A sensitivity analysis including only studies with a low risk of bias, and comparing levosimendan to placebo, phosphodiesterase inhibitors and dobutamine.

OR, odds ratio; I^2, ^heterogeneity

## Discussion

Cardiogenic shock (CS) is a devastating complication of ST-segment myocardial infarction (STEMI) and occurs in 5 to 8% of these patients [12]. Thirty-day survival of STEMI patients in cardiogenic shock is between 40% and 60% [12,13]. Despite favourable trends in the last 30 years, survival to 6 years remains poor and may be as low as 30% [14-16]. This is comparable to many forms of cancer. Therapeutic options for haemodynamic support after coronary revascularisation are limited to pharmacologic or mechanical interventions. Pharmacologic choices are vasopressors and inotropic drugs. Inotropic agents are essential to address the contractile dysfunction that occurs by providing short-term haemodynamic improvement. However, this happens at a cost of increased oxygen demand, arrythmogenesis and potential myocardial injury at a time when the myocardium is most vulnerable. Data on comparison between inotropes are scant and recent reports have highlighted the challenge in selecting the best pharmacologic option [17-19]. Compared with norepinephrine, the use of dopamine in patients with cardiogenic shock has been associated with an increased rate of death [17].

A recent meta-analysis evaluated the use of levosimendan in a variety of patient populations that included post-cardiac surgery, post-vascular surgery, sepsis, decompensated heart failure and post percutaneous coronary intervention (PCI) patients [20]. The proposed mechanisms of myocardial dysfunction in sepsis, after non-cardiac surgery and in the context of cardiogenic shock, are all quite disparate [21,22]. This implies that vasoactive drugs may perform differently, depending on the prevailing mechanism of shock making the interpretation of a pooled analysis of such a heterogeneous group of patients difficult. For this reason we limited this meta-analysis to patients having coronary revascularisation.

Overall, this study shows a mortality benefit when levosimendan was used in patients having coronary revascularisation. The effect was significant in the elective revascularisation group. The subset of patients undergoing emergency revascularisation was under-represented and the mortality benefit in this group was not statistically significant. This latter analysis is probably underpowered. Additionally, levosimendan was associated with favourable outcomes in several clinically important endpoints. The rate of post-revascularisation atrial fibrillation was reduced. Atrial fibrillation after coronary revascularisation is associated with a prolonged hospital stay, increased morbidity and long-term mortality [23]. The analysis also found levosimendan to be associated with a reduction in peri-procedural cardiac troponin I levels. Elevations in cardiac troponin I levels are a valuable marker of myocardial damage after coronary revascularisation and have been associated with a significantly higher rate of in-hospital and long-term mortality [24-27]. The haemodynamic response to levosimendan was also favourable. It is likely that the improvement of haemodynamic profile observed with levosimendan can be sustained for a longer period of time compared to dobutamine or milrinone [28]. This is because levosimendan has a poorly protein-bound active metabolite and may exert clinical effects for up to a week [29], at a much lower energy expenditure than conventional inotropes.

The sub-group analysis comparing levosimendan to the placebo, dobutamine and phoshodiesterase inhibitors all showed a favourable trend towards levosimendan with only the latter group showing statistical significance. This observation could be interpreted as potential harm from exposure to phosphodiesterase inhibitors. Levosimendan reduced the ICU length of stay by a mean of 26.18 hours (95% CI 46.20 to 6.16). The estimated mean cost of critical care is US$3,518/day in the United States and about £1,647/day in the United Kingdom [30,31]. Levosimendan is considerably more expensive than conventional treatment and this probably warrants further pharmaco-economic evaluation.

The findings of this meta-analysis must be viewed in the context of other randomised studies and systematic reviews comparing other vasoactive agents [17,29,32]. Thackray *et al*. systematically reviewed the use of inotropes in cardiac failure [32]. The drugs included were beta agonists, dobutamine, dopexamine and phosphodiesterase inhibitors. Compared with the placebo, these drugs were found to be associated with a non-significant trend for increased rate of death (OR 1.5, 95% CI 0.51 to 3.92).

Our study has several limitations. Several studies were of sub-optimal quality. Eight of the 17 studies included did not have clear allocation concealment. This has the potential to exaggerate treatment effects. Only five studies explicitly stated that the analysis was done by an intention to treat principle. The study by Fuhrmann had a small number of events(i.e. all cause mortality at 30 days) and was stopped early for patient benefit [33]. This study may potentially represent an exaggerated effect size in favour of levosimendan [34]. The sensitivity analysis considering the effect of levosimendan on mortality across comparator drugs showed a more pronounced effect compared with the phosphodiesterase group. Comparing levosimendan to placebo is probably of little value when suitable alternatives exist. These studies recruited low risk patients with a low event rate. Lower event rates require larger sample sizes and it is unsurprising that no statistically significant mortality difference was demonstrated.

Publication bias may account for some of the effects observed. Selective reporting of smaller studies, usually of lower methodological quality will tend to exaggerate treatment effects. The funnel plot was symmetrical, suggesting a low probability of publication bias. Not all studies reported clinically important outcomes. Only 465 patients were included in the analysis of atrial fibrillation rates and only 470 patients were included in the analysis of length of ICU stay.

## Conclusions

This meta-analysis suggests that the use of levosimendan in patients having coronary revascularisation is associated with a significant reduction in mortality. Additionally, the use of levosimendan is also associated with improvement in haemodynamics and reduction in cardiac biomarkers, length of ICU stay and rate of atrial fibrillation. A suitably powered RCT is required to confirm these findings. Additionally, the exact timing and dosing of levosimendan in patients undergoing coronary revascularisation needs to be specifically addressed.

## Key messages

• Acute cardiovascular dysfunction after coronary revascularisation occurs in about 8% to 25% of patients.

• The short-term use of inotropes is crucial to restoring haemodynamics and tissue perfusion though the ideal inotrope in this group of patents is controversial.

• Levosimendan has a potential role in reducing the mortality and morbidity associated with coronary revascularisation.

## Abbreviations

CI: confidence interval; CS: cardiogenic shock; OR: odds ratio; PCI: percutaneous coronary intervention; RR: relative risk; RCT: randomised controlled trials; STEMI: ST-segment myocardial infarction.

## Competing interests

The authors declare that they have no competing interests.

## Authors' contributions

All of the authors contributed to the design of the study. RM and VM were responsible for the statistical analysis. RM drafted the manuscript. All of the authors critically revised the manuscript and agreed on the submitted version.

## Supplementary Material

Additional file 1 **Search strategy for PUBMED**. Click here for file

Additional file 2 **Risk of Bias Table **(e Figure 1). Click here for file
