# Supplementary material for: Levosimendan and mortality after coronary revascularisation: a meta-analysis of randomised controlled trials
Source: Crit Care. 2011 Jun 8;15(3):R140. doi: 10.1186/cc10263 (PMC3219012; doi:10.1186/cc10263)
Supplement: Additional file 1 — Search strategy for PUBMED. [file cc10263-S1.DOC]

Supplementary Material

Appendix 1. Search Strategy for PUBMED.

Terms

# 1 Levosimendan

#2 RANDOMISED CONTROLLED TRIALS

#3 CONTROLLED CLINICAL TRIAL

# 4 RANDOMISED CONTROL TRIAL

#5 RANDOM ALLOCATION

#6 HUMAN

#7 ADULT

Searches

#1

#1 AND #2 OR #3 OR #4 OR #5

#1 AND #6 AND #2 OR #3 OR #4 OR #5

#1 AND #7 AND #2 OR #3 OR #4 OR #5

#1 AND #6 AND #7 AND #2 OR #3 OR #4 OR #5
