# Supplementary material for: Levosimendan and mortality after coronary revascularisation: a meta-analysis of randomised controlled trials
Source: Crit Care. 2011 Jun 8;15(3):R140. doi: 10.1186/cc10263 (PMC3219012; doi:10.1186/cc10263)
Supplement: Additional file 2 — Risk of Bias Table (e Figure 1). [file cc10263-S2.DOCX]

Appendix 2. Risk of Bias Graph (eFigure 1)


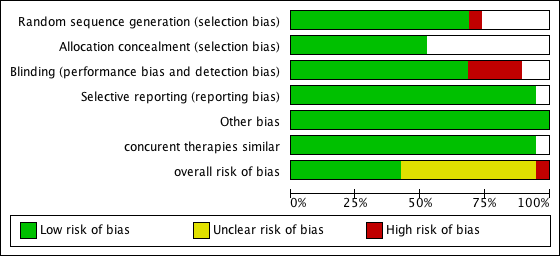


eFigure 1. Risk of bias graph: review authors' judgements about each risk of bias item presented as percentages across all included studies
